# CD38^+^CD39^+^ NK cells associate with HIV disease progression and negatively regulate T cell proliferation

**DOI:** 10.3389/fimmu.2022.946871

**Published:** 2022-10-04

**Authors:** Shi Qian, Chunbin Xiong, Meiting Wang, Zining Zhang, Yajing Fu, Qinghai Hu, Haibo Ding, Xiaoxu Han, Hong Shang, Yongjun Jiang

**Affiliations:** ^1^ National Health Commission (NHC) Key Laboratory of AIDS Immunology (China Medical University), National Clinical Research Center for Laboratory Medicine, The First Hospital of China Medical University, Shenyang, China; ^2^ Key Laboratory of AIDS Immunology, Chinese Academy of Medical Sciences, Shenyang, China; ^3^ Department of Clinical Laboratory, Tianjin Medical University General Hospital, Tianjin, China; ^4^ Units of Medical Laboratory, Chinese Academy of Medical Sciences, Shenyang, China

**Keywords:** HIV, NK cells, CD38, CD39, negative regulation

## Abstract

The ectonucleotidases CD38 and CD39 have a critical regulatory effect on tumors and viral infections *via* the adenosine axis. Natural killer (NK) cells produce cytokines, induce cytotoxic responses against viral infection, and acquire immunoregulatory properties. However, the roles of CD38 and CD39 expressed NK cells in HIV disease require elucidation. Our study showed that the proportions of CD38^+^CD39^+^ NK cells in HIV-infected individuals were positively associated with HIV viral loads and negatively associated with the CD4^+^ T cell count. Furthermore, CD38^+^CD39^+^ NK cells expressed additional inhibitory receptors, TIM-3 and LAG-3, and produced more TGF-β. Moreover, autologous NK cells suppressed the proliferation of CD8^+^ T and CD4^+^ T cells of HIV-infected individuals, and inhibiting CD38 and CD39 on NK cells restored CD8^+^ T and CD4^+^ T cell proliferation *in vitro*. In conclusion, these data support a critical role for CD38 and CD39 on NK cells in HIV infection and targeting CD38 and CD39 on NK cells may be a potential therapeutic strategy against HIV infection.

## Introduction

NK cells are crucial types of immunocytes. They kill cancerous and virus-infected cells (1) while negatively regulating the immune system (2). Previous research has revealed that NK cells can alleviate inflammation during systemic infections by producing interleukin (IL)-10 (3); while one of the NK cell subsets in HIV-infected individuals, CD56−CD16+, negatively regulated IFN-γ release by CD8+ T cells (4).

Extracellular enzymes on immune cells regulate the balance between the proinflammatory extracellular adenosine triphosphate (ATP) and immunosuppressive extracellular adenosine in the tumor microenvironment (5). Ectonucleotidases, such as CD39, CD38, and CD73, are involved in immune modulation (6). Ectoenzyme CD39 hydrolyzes extracellular ATP and adenosine diphosphate (ADP) to generate adenosine monophosphate (AMP) (7). Moreover, CD38 transforms the nicotinamide adenine dinucleotide (NAD+) into ADP-ribose (ADPR), which is converted to AMP by the ectonucleotide pyrophosphatase/phosphodiesterase 1 (ENPP1) (8–10). With the participation of CD73, AMP is converted to adenosine, which inhibits the function of immune cells (9, 11). In the immune system, the cell types that express CD39 include B cells, monocytes, NK cells, and T cells (12). In non-tumor populations, CD38 is generally expressed on NK cells, dendritic cells, B cells, plasma cells, and T cells (10, 13). Previous studies have shown that Tregs expressing CD39 inhibited the antitumor immunity mediated by NK cells (14) and that anti-CD38 antibodies decreased Tregs’ suppression and restored CD4+CD25- T cell proliferation in multiple myeloma by decreasing the percentage of CD38 expression (15). Multiple myeloma cells with high CD38 expression reduce the number and activity of NK cells (16). CD56brightCD16− NK cells from blood of juvenile idiopathic arthritis patients highly expressed CD38 molecules, and CD56brightCD16− NK cells inhibited autologous CD4+ T cell proliferation via the CD38-mediated pathway (17).

The CD38 molecule is a marker of activation. The proportion of CD38+ NK cells in HIV infection is increased and positively or negatively associated with the HIV viral load and CD4+ T cell count, respectively (18–20). CD39, an enzyme involved in extracellular nucleotide metabolism, is highly expressed on CD56bright NK cells and is tightly correlated with viral load and to CD4+ T cell count in untreated individuals infected with HIV. However, because CD39 and CD38 are extracellular nucleotide enzymes and produce AMP via different pathways, the correlation between the CD38 and CD39 dual-positive NK cells and disease progression is still unclear. Moreover, the characteristics, cytokine production, and regulation of CD39+CD38+ NK cells need to be elucidated.

Here, we investigated the expression of CD38 and CD39 on NK cells and analyzed the correlation between CD39+CD38+ NK cells and disease progression (HIV viral load and CD4+ T cell count). Furthermore, we explored whether the expression of CD39 and CD38 on NK cells could be related to inhibitory receptors that modulate CD4+ T and CD8+ T cell proliferation. Our findings reveal that the immunosuppression mediated via CD39 and CD38 on NK cells is crucial in modulating CD4+ T and CD8+ T cell proliferation in HIV infection.

## Materials and methods

### Research participants

Research participants infected with HIV were enrolled in the First Hospital of China Medical University, including 25 untreated individuals infected with HIV (untreated HIV) and 70 individuals infected with HIV receiving antiretroviral therapy (ART). The 20 healthy controls (HC) who were not infected with hepatitis B or C were HIV-negative. Details of the participants in this study are shown in **Table 1**. Informed consent was collected from study subjects. The study protocol was approved by the Research and Ethics Committee of The First Hospital of China Medical University and was performed in accordance with the Declaration of Helsinki.

**Table 1:** Clinical characteristics of subjects enrolled in this study.

Characteristics	HC	Untreated HIV	ART	*p*-value
Number	20	25	70	
Gender (male/female)	9/11	24/1	66/4	
Age(years)	38.50(24.00,47.00)	29.00(26.00,31.00)	32.00(28.00,37.00)	> 0.05
CD4^+^ T cell count (cells/μL)	NA	350.00(272.00,545.00)	611.50(474.00,821.75)	< 0.05
Viral load (Lg copies/mL)	NA	4.44 (3.90,4.58)	undetectable	

Quantitative data are expressed as the median (interquartile range).

NA, not available.

### Detection of CD38, CD39, and expression of inhibitory receptors

Fresh PBMC were obtained from human blood samples. Human TruStain FcX™ (BioLegend, USA) was added for 5 mins to block Fc receptors. Cell surface staining antibodies included: anti-CD3-fluorescein isothiocyanate (FITC), or Percp, anti-CD56-phycoerythrin (PE)-Cyanin7 (Cy7), anti-CD16-BV786 or BV510, anti-CD14-Percp, anti-CD19-Percp, anti-CD4-Allophycocyanin (APC)-Cy7, anti-CD39-PE or APC, and anti-CD38-BV421 or FITC, anti-TIGIT-BV421, anti-TIM-3-PE, anti-LAG-3-AF647, anti-PD-L1-BV421(BioLegend, USA). 7-AAD was applied to detect dead cells. Cells were measured by an LSR II Fortessa cytometer (BD Biosciences, USA). For the stimulation experiment of CD38 expression, IL-12 (10 ng/mL, R&D, USA), IL-15 (50 ng/mL, R&D,USA), and IL-18 (100 ng/mL, R&D,USA) were used to stimulate PBMC. The PBMC was cultured for 72 h and stained as indicated above.

### Measurement of interleukin-10 and TGF-β production

An isolation kit (Stemcell, CAN) was used to isolate the NK cells, which were activated by Phorbol 12-myristate 13-acetate (PMA)/ionomycin (BioLegend, USA) and protein transport inhibitor GolgiStop (BD Biosciences) in 96-well U-bottomed plates with 5% CO2 at 37 °C for 6 h. First, the NK cells were collected, and Fixable viability stain 620 (BD Pharmingen, USA) was added to exclude dead cells. Antibodies for cell surface staining included anti-CD3-APC-CY7, anti-CD56-PE-Cy7, anti-CD38-PE, anti-CD39-FITC (BioLegend, USA) and were incubated for 20 mins. Next, the cells were fixed and washed by Fixation/Permeabilization solution (BD Biosciences, USA) and intracellularly labeled by anti-IL-10-APC and anti-TGF-β-BV421 (BD Biosciences, USA) at 4 °C for 20 mins. Finally, the cells were measured by LSR II Fortessa cytometer (BD Biosciences, USA).

### Detection of the proliferation of T cells suppressed by NK cells

NK, CD4+ T, and CD8+ T cells were collected using negative isolation kits (Stemcell, CAN). The NK cells were cultured with a CD38 inhibitor 1 (78c, 0.5 μM, MCE, USA), ARL67156 trisodium salt (100 μM, MCE, USA), AB-680 (100 nM, MCE, USA), and EHNA hydrochloride (100 μM, MCE,USA) for 30 mins. CD4+ T and CD8+ T cells were stained using CellTrace Violet (Invitrogen, USA) for 30 mins. The NK cells and CD4+ T or CD8+ T cells were activated by Dynabeads™ Human T-Activator CD3/28 (Gibco, USA) and incubated at a ratio of 1:1 for 3 days. The cells were collected, washed, and dyed with anti-CD4-APC-CY7 or anti-CD8-FITC. The dead cells were detected using 7-AAD. The samples were detected by the LSR II Fortessa cytometer (BD Biosciences, USA).

### Statistical analysis

Flow cytometry data were analyzed using FlowJo™ 10.5.0. All statistical analyses were conducted using GraphPad Prism v9.0.0. Comparisons of data in multiple groups, unpaired groups, and paired groups were performed using the Kruskal–Wallis test, the Mann–Whitney U test, and the Wilcoxon matched-pairs signed-rank test, respectively. Correlations were analyzed by the Spearman’s rank test; a P-value < 0.05 was considered a significant difference.

## Results

### Higher proportions of CD39 and CD38 on NK cells in HIV infected individuals

**Figure 1:**
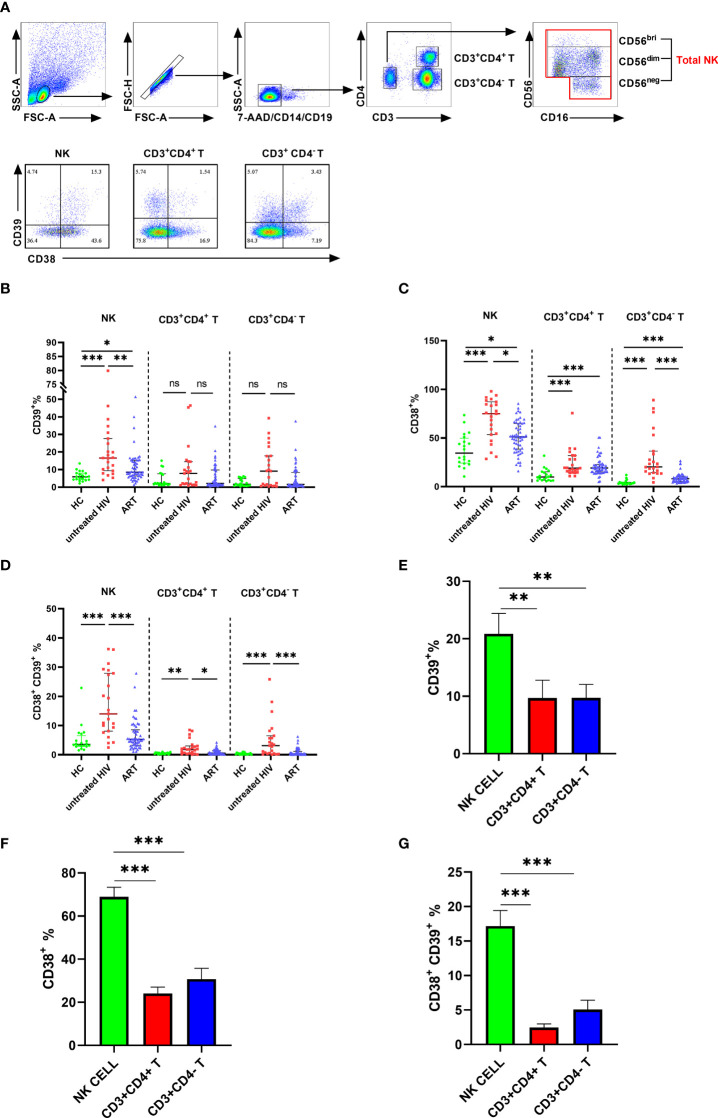
The expression of CD38 and CD39 on NK, CD3+CD4- T and CD3+CD4- T cells in HC, untreated HIV, and ART groups. **(A)** Gating strategy from flow cytometry analysis and the representative dot plots showing the expression of CD38 and CD39 on NK, CD3^+^CD4^+^ T cells, and CD3^+^CD4^-^ T cells. **(B)** Comparison of the expression of CD39 on NK, CD3^+^CD4^+^ T, and CD3^+^CD4^-^ T cells in HC, untreated HIV, and ART groups. **(C)** Comparison of the expression of CD38 on NK, CD3^+^CD4^+^ T and CD3^+^CD4^-^ T cells in HC, untreated HIV, and ART groups. **(D)** Comparison of the co-expression of CD38 and CD39 on NK, CD3^+^CD4^+^ T, and CD3^+^CD4^-^ T cells in HC, untreated HIV and ART groups. **(E)** Comparison of the expression of CD39 on NK, CD3^+^CD4^+^ T, and CD3^+^CD4^-^ T cells in HIV. **(F)** Comparison of the expression of CD38 on NK, CD3^+^CD4^+^ T, and CD3^+^CD4^-^ T cells in HIV. **(G)** Comparison of the co-expression of CD38 and CD39 on NK, CD3^+^CD4^+^ T, and CD3^+^CD4^-^ T cells in HIV. The Kruskal–Wallis test was used to compare the three groups. *p < 0.05, **p < 0.01, ***p < 0.001, ns: no significance.

The expression of CD39 and CD38 on NK cells and T cells was measured in individuals infected with HIV and healthy donors to investigate the changes in CD39 and CD38 expression after HIV infection. The NK cells were distinguished by CD3- CD14- CD19- CD16+/CD56+, and the subsets of T cells were distinguished by CD3+ CD4+ or CD3+ CD4- (**Figure 1A**). Compared with a heathy control (HC) group, the proportion of CD39+ NK cells was increased in the untreated HIV group (P < 0.001), and the proportion of CD39+ NK cells in the untreated HIV group was higher than that in the ART group (P < 0.01; **Figure 1B**). In the untreated HIV group, CD38 expression on NK cells was higher than HC group (P < 0.001; **Figure 1C**), and the CD38 expressed on the NK cells in the ART group was lower than the untreated HIV group (P < 0.05; **Figure 1C**). As expected, the proportion of CD38+CD39+ NK cells was higher in the untreated HIV group (P < 0.001; **Figure 1D**). HIV infection was conducive to the upregulation of CD38 (P < 0.001; **Figure 1C**), but not to CD39 levels on CD3+ CD4+ and CD3+ CD4- T cells (P > 0.05; **Figure 1B**). The CD39 and CD38 expression on NK cell were of great importance. Furthermore, the proportions of CD73+ NK cells in the untreated HIV group tended to increase compared with the HC group (**Supplementary Figure S1A**). In lymphocytes, a minimal proportion of CD3+CD4+ and CD3+CD4- T cells express CD39 compared to the NK cells from the individuals infected with HIV (P < 0.01 for CD4+ T and P < 0.01 for CD4- T; **Figure 1E**). Similarly, CD38 is expressed less frequently on CD3+CD4+ and CD3+CD4- T cells than on NK cells from the individuals infected with HIV (P < 0.001 for CD4+ T and P < 0.001 for CD4- T; **Figure 1F**). The coexpression of CD38 and CD39 was higher on NK cells than on CD3+CD4+ and CD3+CD4- T cells in individuals infected with HIV (P < 0.001 for CD4+ T and P < 0.001 for CD4- T; **Figure 1G**). These findings indicated that NK cells expressed more ectonucleotidases than CD3+CD4+ or CD3+CD4- T cells in individuals infected with HIV.

### The expression of CD39 and CD38 on NK subsets

**Figure 2:**
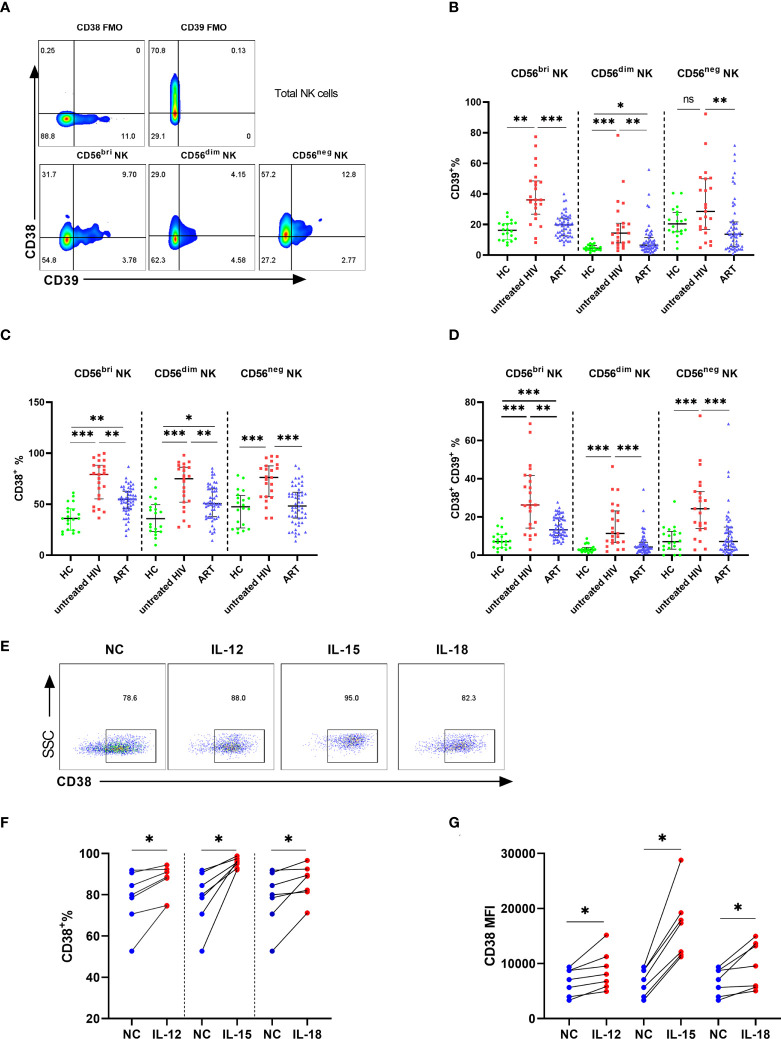
The expression of CD39 and CD38 on NK subsets. **(A)** The fluorescence minus one (FMO) control of CD38/CD39 on total NK cells and representative flow cytometry plots showing three subsets of NK cells. **(B)** Comparison of the expression of CD39 on CD56^bri^, CD56^dim^, and CD56^neg^ NK cells in HC, untreated HIV, and ART groups. **(C)** Comparison of the expression of CD38 on CD56^bri^, CD56^dim^ and CD56^neg^ NK cells in HC, untreated HIV and ART groups. **(D)** Comparison of the co-expression of CD38 and CD39 on CD56^bri^, CD56^dim^, and CD56^neg^ NK cells in HC, untreated HIV and ART groups. **(E)** The representative flow cytometry dot plots of CD38 expression on NK cells stimulated by IL-12/15/18. **(F)** Comparison of the percentage of CD38^+^ NK cells from HIV seropositive individuals in the negative control (NC) group and NK cells stimulated with IL-12, IL-15, and IL-18, respectively. **(G)** The MFI of CD38 on NK cells from HIV infected individuals between negative control (NC) group and the group stimulated with IL-12, IL-15, and IL-18, respectively. *p < 0.05, **p < 0.01, ***p < 0.001, ns: no significance.

Next, we investigated the proportions of CD39 and CD38 in the subpopulation of NK cells. NK cells were classified into three subpopulations according to the level of CD56 and CD16 (**Figure 2A**). The untreated HIV group had a higher proportion of CD39 on CD56bright and CD56dim NK cells than that of controls (P < 0.01 for CD56bright NK and P < 0.001 for CD56dim NK). However, CD39 expression was showed no significance differences between the untreated HIV group and the HC group on the CD56neg NK cells (**Figure 2B**), which indicated that the difference in CD39 expression derived from the CD56bri and CD56dim NK cells. Higher CD38 levels were found in all the NK cell subpopulations in the untreated HIV group compared with controls (P < 0.001 for CD56bright NK, P < 0.001 for CD56dim NK and P < 0.001 for CD56neg NK; **Figure 2C**). The proportion of the CD38+CD39+ subset in the untreated HIV group was also increased in all subpopulations (P < 0.001 for CD56bright NK, P < 0.001 for CD56dim NK and P < 0.001 for CD56neg NK; **Figure 2D**). In previous research, the expression of CD39 on CD56bright NK cells was induced by cytokines (21). PBMC from HIV subjects were evaluated after stimulation with IL-12, IL-15, and IL-18 to explore the increased in CD38+ NK cells. The proportion of CD38+ NK cells were markedly increased after stimulation (P < 0.05 for IL-12, P < 0.05 for IL-15 and P < 0.05 for IL-18; **Figures 2E, F**). Similarly, the mean fluorescence intensity (MFI) of CD38 on the NK cells increased more than the negative control (P < 0.05 for IL-12, P < 0.05 for IL-15 and P < 0.05 for IL-18; **Figure 2G**).

### The proportion of CD38+CD39+ NK cells was positively associated with HIV disease progression

**Figure 3:**
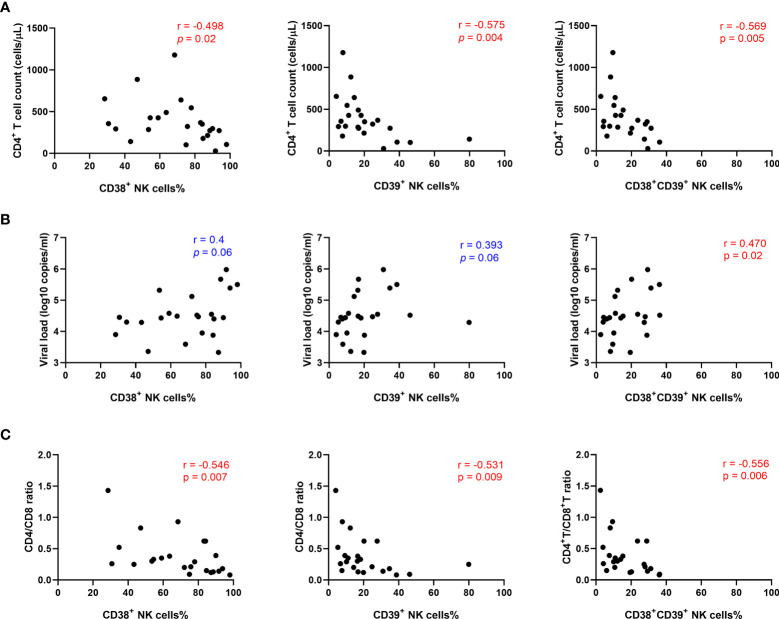
The percentage of CD38+CD39+ NK cells was positively associated with HIV disease progression. **(A)** Association between the CD4^+^ T cell count and the expression of CD38^+^, CD39^+^, and CD38^+^39^+^ NK cells in untreated HIV infection individuals. **(B)** Correlations between the viral load and the expression of CD38^+^, CD39^+^, and CD38^+^39^+^ NK cells in untreated HIV infection individuals. **(C)** Correlations between the CD4/CD8 ratio and the expression of CD38^+^, CD39^+^, and CD38^+^39^+^ NK cells in untreated HIV infection individuals.

To evaluate whether higher levels of CD38 and CD39 were associated with disease progression, correlation analyses were performed in the untreated HIV group. The results showed that the levels of CD38+, CD39+, and CD38+CD39+ NK cells were negatively correlated with the CD4+ T cell count (r = -0.498, P = 0.02; r = -0.575, P= 0.004; r = -0.569, P = 0.005, respectively; **Figure 3A**). Furthermore, viral load was positively associated with the frequencies of the CD38+ CD39+ NK cells (r = 0.470, P = 0.02; **Figure 3B**), but not the CD38+ and the CD39+ NK cells (r =0.4, P = 0.06; r = 0.393, P = 0.06; **Figure 3B**). Moreover, the proportions of the CD38+, CD39+, and CD38+CD39+ NK cells were negatively correlated with the CD4+/CD8+ T cell count ratio. (r = -0.546, P = 0.007; r = -0.531, P = 0.009; r = -0.556, P = 0.006, respectively; **Figure 3C**).

### The expression of inhibitory receptors increased on CD38+ and/or CD39+ NK cells

**Figure 4:**
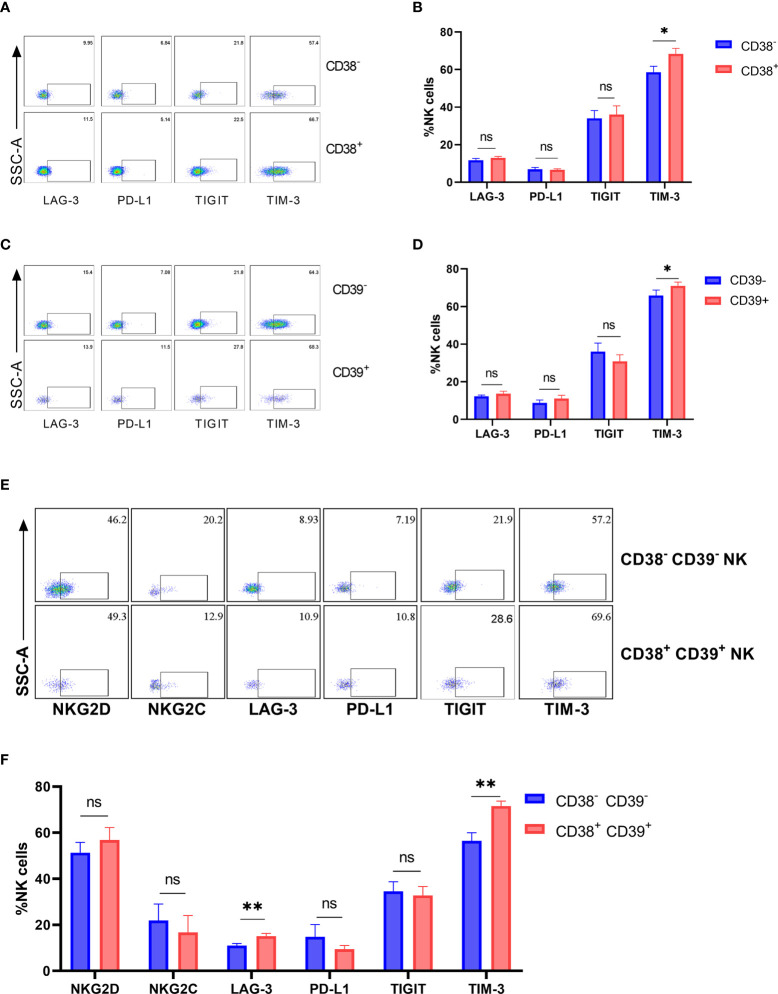
The expression of inhibitory receptors increased on CD38+ or/and CD39+ NK cells. **(A)** The representative flow cytometry plots of LAG-3, PD-L1, TIGIT, and TIM-3 on CD38^-^ and CD38^+^ NK cells. **(B)** Comparison of the expression of LAG-3, PD-L1, TIGIT, and TIM-3 between CD38^-^ and CD38^+^ NK cells. **(C)** The representative flow cytometry plots of LAG-3, PD-L1, TIGIT, and TIM-3 on CD39^-^ and CD39^+^ NK cells. **(D)** Comparison of the expression of LAG-3, PD-L1, TIGIT, and TIM-3 between CD39^-^ and CD39^+^ NK cells. **(E)** The representative flow cytometry plots of NKG2D, NKG2C, LAG-3, PD-L1, TIGIT, and TIM-3 on CD38^-^CD39^-^ and CD38^+^CD39^+^ NK cells. **(F)** Comparison of the expression of NKG2D, NKG2C, LAG-3, PD-L1, TIGIT, and TIM-3 on CD38^-^CD39^-^ and CD38^+^CD39^+^ NK cells. *p < 0.05, **p < 0.01, ns: no significance.

The inhibitory receptors (LAG-3, PD-L1, TIGIT, and TIM-3) were detected on the CD38+, CD39+, and CD38+CD39+ NK cells from the untreated HIV group to determine whether the inhibitory receptor increased on CD38+CD39+ NK cells. No obvious differences were found in the expressions of LAG-3, PD-L1, and TIGIT between negative and positive subsets (**Figures 4A–D**). The proportion of TIM-3 on the CD38+ NK cells was greater than the CD38- NK cells (P < 0.05, **Figure 4B**). Similarly, the CD39+ NK cells expressed higher levels of TIM-3 than the CD39- NK cells (P < 0.05, **Figure 4D**). Moreover, the expression of active receptors (NKG2D and NKG2C) between the CD38+CD39+ and CD38-CD39-NK cells were measured (**Figure 4E**), but no obvious differences were found (P > 0.05 for NKG2D and P > 0.05 for NKG2C; **Figure 4F**). With regard to the inhibitory receptors, the expression of LAG-3 and TIM-3 on the CD38+ CD39+ NK cells was greater than on CD38-CD39- NK cells (P < 0.01 for LAG-3 and P < 0.01 for TIM-3; **Figure 4F**). A higher level of inhibitor receptors of the CD38+CD39+ NK cells indicated that these subsets may be associated with negative cell function.

### The proportion of TGF-β and interleukin-10 on CD38+ or/and CD39+ NK cells

**Figure 5:**
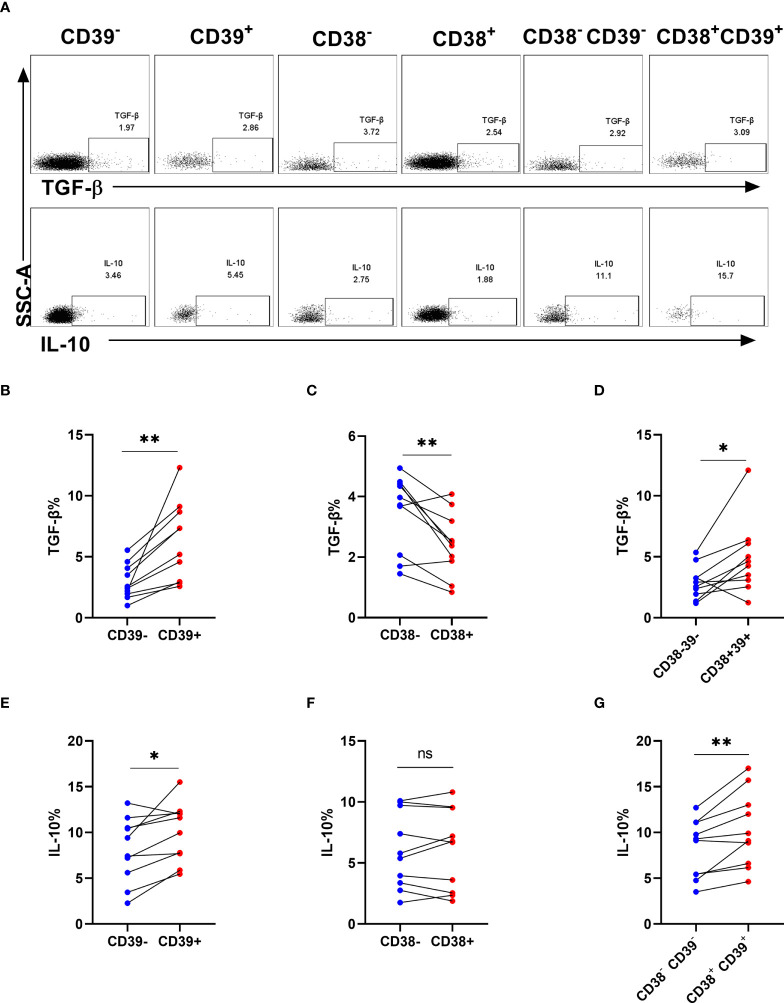
The proportion of TGF-β and interleukin-10 on CD38+ or/and CD39+ NK cells. **(A)** The representative flow cytometry spots showing the production of TGF-β and IL-10 in CD39^-^, CD39^+^, CD38^-^, CD38^+^, CD38^-^CD39^-^, and CD38^+^CD39^+^ NK cells from HIV infected individuals. **(B–D)** Comparison of the production of TGF-β between CD39^-^ and CD39^+^ NK cells **(B)**, CD38^-^ and CD38^+^ NK cells **(C)**, CD38^-^CD39^-^ and CD38^+^CD39^+^ NK cells **(D)** from HIV infected participants stimulated with PMA/ionomycin for 6 h. **(E–G)** Comparison of the production of IL-10 between CD39^-^ and CD39^+^ NK cells **(E)**, CD38^-^ and CD38^+^ NK cells **(F)**, CD38^-^CD39^-^ and CD38^+^CD39^+^ NK cells **(G)** from HIV infected participants stimulated with PMA/ionomycin for 6 h. *p < 0.05, **p < 0.01, ns: no significance.

To evaluate the cell function in producing cytokines, we analyzed the frequencies of TGF-β and IL-10 in NK cells after stimulation (**Figure 5A**). Interestingly, TGF-β and IL-10 expression was higher in CD39+ NK cells than CD39- NK cells (P < 0.01 for TGF-β and P < 0.05 for IL-10, **Figures 5B, E**). CD38+ NK cells did not express more TGF-β and IL-10 than CD38- NK cells (P < 0.05 for TGF- β and P> 0.05 for IL-10, **Figures 5C, F**). Furthermore, TGF-β and IL-10 expression was upregulated in CD38+CD39+ NK compared to CD38-CD39- NK cells (P < 0.05 for TGF-β and P < 0.01 for IL-10, **Figures 5D, G**).

### NK cells suppressed the proliferative ability of CD4+ T and CD8+ T cells in individuals infected with HIV while CD38, CD39, and CD73 inhibitors reverse the effect

**Figure 6:**
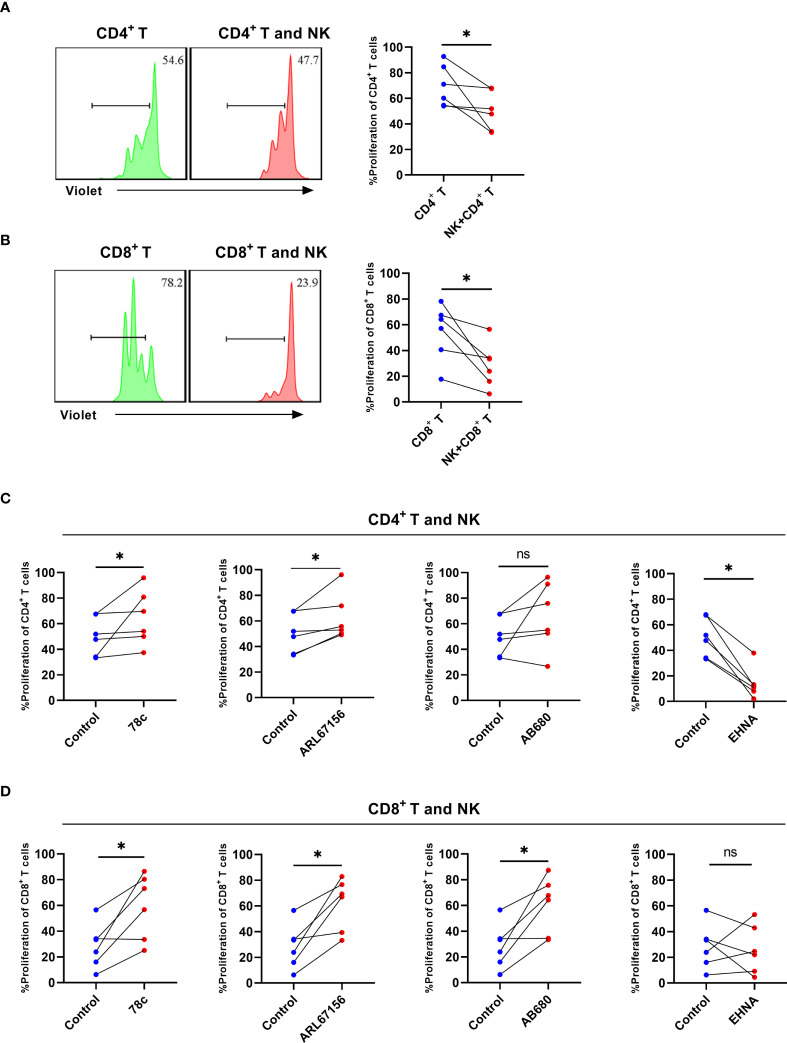
NK cells from HIV infected individuals inhibit the proliferation of CD4^+^ T and CD8^+^ T cells and CD38, CD39, and CD73 inhibitors reverse the effect. **(A)** A representative histogram and comparison showing the proliferation of CD4^+^ T cells with or without autologous NK cells for 3 days. **(B)** A representative histogram and comparison showing the proliferation of CD8^+^ T cells with or without autologous NK cells for 3 days. **(C)** Comparison of the proliferation of CD4^+^ T cells cultured with NK cells from untreated HIV infected individuals stimulated with 78c, ARL67156, AB680, and EHNA. **(D)** Comparison of the proliferation of CD8^+^ T cells cultured with NK cells from untreated HIV infected individuals stimulated with 78c, ARL67156, AB680 and EHNA. *p < 0.05, ns: no significance.

To assess the role of CD38 and CD39 on NK cells in T cell proliferation, NK cells from the untreated HIV group were cultured with CD4+ T or CD8+ T cells, considering that the NK cells of the untreated HIV group expressed higher levels of CD38 and CD39. The NK cells decreased the proliferation of CD4+ T and CD8+ T cells (P < 0.05 for CD4+ T and P < 0.05 for CD8+ T; **Figures 6A, B**). Consequently, CD38 and CD39 expression on NK cells was inhibited before being cultured with CD4+ T or CD8+ T cells. The CD38 inhibitor, 78c, restored the proliferation of the CD4+ T and CD8+ T cells (P < 0.05 for CD4+ T and P < 0.05 for CD8+ T, **Figures 6C, D**). Similarly, the proliferation of CD4+ T and CD8+ T cells was restored by ARL67156, a potent inhibitor of CD39 (22) (P< 0.05 for CD4+ T and P <0.05 for CD8+ T) (**Figures 6C, D**). The CD73 inhibitor, AB680, also restored the proliferation of CD8+ T cells and induced CD4+ T cells proliferation (P> 0.05 for CD4+ T and P <0.05 for CD8+ T) (**Figures 6C, D**). Next, to further support our hypothesis, we inhibited adenosine deaminase (ADA) that converts adenosine to inosine treatment with EHNA (23, 24). Our findings indicated that the proliferation of CD4+ T cells was impaired after inhibiting ADA (P< 0.05, **Figure 6C**), while the proliferation of CD8+ T cells was not affected (P > 0.05, **Figure 6D**).

## Discussion

Ectonucleotidases, including CD38, CD39, and CD73, have a vital role in the treatment and prognosis of autoimmunity and viral infection (25). In this study, we found that CD38+CD39+ NK cells were more abundant in the untreated HIV group, and that this increase correlated with HIV disease progression. Furthermore, CD38 and CD39 on NK cells in the HIV group reduced the proliferation of the CD4+ T and CD8+ T cells by promoting adenosine production. In addition, in vitro inhibition of CD38 and CD39 expression on NK cells restored autologous CD4+ and CD8+ T cell proliferation.

In this study, we found a higher proportion of CD39 and CD38 on NK cells in the untreated HIV group. Although Dierks et al. (21) also reported higher proportions of CD39+ NK cells in individuals infected with HIV, which may be related to the pathogenesis of HIV, the mechanism was not further explored. To further understand the characteristics of NK cells expressing ectonucleotidases, NK cells expressing CD38 or/and CD39 were detected, and the proportion of CD38+CD39+ NK cells increased in HIV infected subjects. An early study suggested that upregulated levels of CD39 on NK cells were associated with immune activation in HIV infection, while the proinflammatory factors (IL-12, IL-15, and IL-18) upregulated the CD39 expression on NK cells (21). In addition, IL-12 and IL-18 stimulate other activation receptors on NK cells in tumors (26). Based on the earlier study, we found that IL-12, IL-15, and IL-18 upregulated the levels of CD38 on NK cells. The mechanism for this remains unknown; however, it may be associated with the activation of NK cells (27). Swaminathan et al. demonstrated that the concentration of IL-15 and IL-18 were upregulated in individuals infected with HIV (28, 29). Another study found that antiretroviral therapy reduced chronic inflammation in these patients by decreasing IL-12 levels (30). Similarly, CD38 and CD39 expression on NK cells decreased after antiretroviral therapy. The persistent chronic inflammation in individuals infected with HIV induced the expression of CD38 and CD39 on NK cells.

Because the ectonucleotidases on NK cells decreased significantly after antiretroviral treatment, we hypothesized that the NK cell ectonucleotidases had a crucial role in the progression of AIDS. Our data suggest that the CD4+ T cell count and CD4/CD8 ratio were negatively associated with the level of CD38 and CD39 on NK cells. Thus, CD38 and CD39 may be associated with the regulation of T cell counts. Interestingly, our data showed that CD38+CD39+ NK cells were negatively related with the CD4/CD8 ratio count and CD4+ T cell and positively related to the HIV viral load. We found that CD38+CD39+ NK cells may negatively regulate antiviral activity. Brauneck et al. (31) suggested that blocking CD39 on NK cells enhanced NK-92 cell-mediated cytotoxicity. Adenosine from the CD39 and CD38 enzymatic cascade is an important immunosuppression pathway in the antiviral function of NK cells (10, 32). Furthermore, CD38+CD39+ NK cells expressed more inhibitory receptors. A possible explanation may be that CD38 and CD39 promoted adenosine production, which had previously been reported to upregulate the level of inhibitory receptors on immune cells (33).

Researchers have found that IL-10 and TGF-β are upregulated in plasma during HIV infection (34, 35). NK cells can secret the cytokines IL-10 and TGF-β, which have been shown to limit the function of T cells (4, 36). Our results showed that CD39+ and CD38+CD39+ NK cells had higher expression of TGF-β. Neo et al. found that NK cells expressing the ectonucleotidases, CD73, produced more IL-10 and TGF-β via STAT3 transcriptional activity (37). In addition, CD39+ Tregs secreted more IL-10 and TGF-β to inhibit inflammation (38). These data indicated that CD38+CD39+ NK cells expressed more IL-10 and TGF-β for immune suppression.

Herein, we showed that autologous NK cells markedly suppressed the proliferation of CD4+ T and CD8+ T cells from untreated individuals with HIV. Morandi et al. demonstrated that adenosine produced by NK cells from inflammatory pleural effusions inhibits the autologous CD4+ T cell proliferation through a CD38-mediated pathway (17). In term of suppressive mechanism, Tomasz et al. found that ATP is released and transformed into adenosine through CD39 and CD73 and play a suppressive role by the adenosine and the A2A receptor, and the activation of A2A receptor reduce the calcium-influx into T cells by inhibiting the tyrosine phosphorylation of the key kinase ZAP-70 (39, 40). In a previous study, CD39+CD73+ tumor cells reduced the proliferation of CD4+ T and CD8+ T cells by CD39 and the adenosine dependent pathway, and treatment with CD39 inhibitors or blocking antibodies reduced the tumor-induced inhibition (41). Julia et al. demonstrated that degradation of extracellular ATP or AMP by ectonucleotidases lead to the accumulation of deoxyATP, which prevent DNA synthesis by inhibiting ribonucleotide reductase (42). In subsequent experiments, inhibitors of CD39, CD38, and CD73 were added to reduce adenosine production and restore T cell proliferation (43). Damaged and stressed cells release NAD+, which are hydrolyzed in a stepwise manner to synthesize adenosine by CD38 and CD73 (44). Furthermore, TCR stimulation induces ATP release, which allows extracellular ATP to act on the P2X7 receptor to promote T cell activation by mediating Ca2+ influx, while CD39 and CD73 suppressed the proliferation of T cells by decreasing ATP and increasing adenosine production (42, 45). The adenosine axis contains CD38, CD39, and CD73 and is a vital immunosuppression pathway. CD8+ T cell dysfunction in chronic HIV infection can be reversed by blocking the CD39/adenosine pathway (46). In addition, CD8+ T cells participate in immune suppression by CD73-mediated adenosine production (47). For NK cells, CD73 defines a population of NK cells with immune modulation properties in the tumor microenvironment (37).

In conclusion, we demonstrated that CD38+CD39+ NK cells are significantly increased in the untreated HIV group and associated with disease progression. Furthermore, the CD38+CD39+ NK cells expressed a higher percentage of inhibitory receptors (TIM-3 and LAG-3) and produced more TGF-β. CD38 and CD39 on NK cells impaired the proliferation of CD4+ T and CD8+ T cells by adenosine-mediated immune modulation. Altogether these findings indicate that the inhibition of CD38 and CD39 on NK cells in individuals with HIV can restore T cell proliferation, which represents a potential immunotherapy target for HIV treatment.

## Limitations

There are some limitations in our study. We demonstrated CD38 and CD39 on NK cells impaired the proliferation of CD4+ T and CD8+ T cells by adenosine-mediated immune modulation, while we did not carry out further mechanistic experiments. There will be opportunities to explore more mechanism about the suppressive function of CD38 and CD39 on NK cells in our future studies, although the mechanism about the suppressive function of CD38 and CD39 have been reported in previous studies as mentioned above in the discussion (39, 41).

## Data availability statement

The original contributions presented in the study are included in the article/**Supplementary Material**. Further inquiries can be directed to the corresponding authors.

## Ethics statement

The studies involving human participants were reviewed and approved by Research and Ethics Committee of The First Hospital of China Medical University. The patients/participants provided their written informed consent to participate in this study.

## Author contributions

SQ, CX conducted the study, statistical analysis and wrote the manuscript. YJ and HS designed the experiments and revised the manuscript. MW, ZZ, YF, QH, XH, and HD recruited participants and provided the clinical data. All authors read and approved the final manuscript.

## Acknowledgments

We express our gratitude to all participants including patients and blood donors in our study. We are appreciated the support from the CAMS Innovation Fund for Medical Sciences(2019-I2M-5-027) and the Liaoning Provincial Department of Education Basic Scientific Research Project (LJKZ0737), Mega-Projects of National Science Research for the 13th Five-Year Plan (2017ZX10201101 and 2018ZX10732101-001-011).

## Conflict of interest

The authors declare that the research was conducted in the absence of any commercial or financial relationships that could be construed as a potential conflict of interest.

## Publisher’s note

All claims expressed in this article are solely those of the authors and do not necessarily represent those of their affiliated organizations, or those of the publisher, the editors and the reviewers. Any product that may be evaluated in this article, or claim that may be made by its manufacturer, is not guaranteed or endorsed by the publisher.

## Supplementary material

Supplementary Figure 1 The expression of CD73 on NK, CD3^+^CD4^-^ T and CD3^+^CD4^-^ T cells in HC, untreated HIV, and ART-treated patients.
