# Supplementary figures and images for: CD38+CD39+ NK cells associate with HIV disease progression and negatively regulate T cell proliferation
Source: Front Immunol. 2022 Oct 4;13:946871. doi: 10.3389/fimmu.2022.946871 (PMC9577302; doi:10.3389/fimmu.2022.946871)

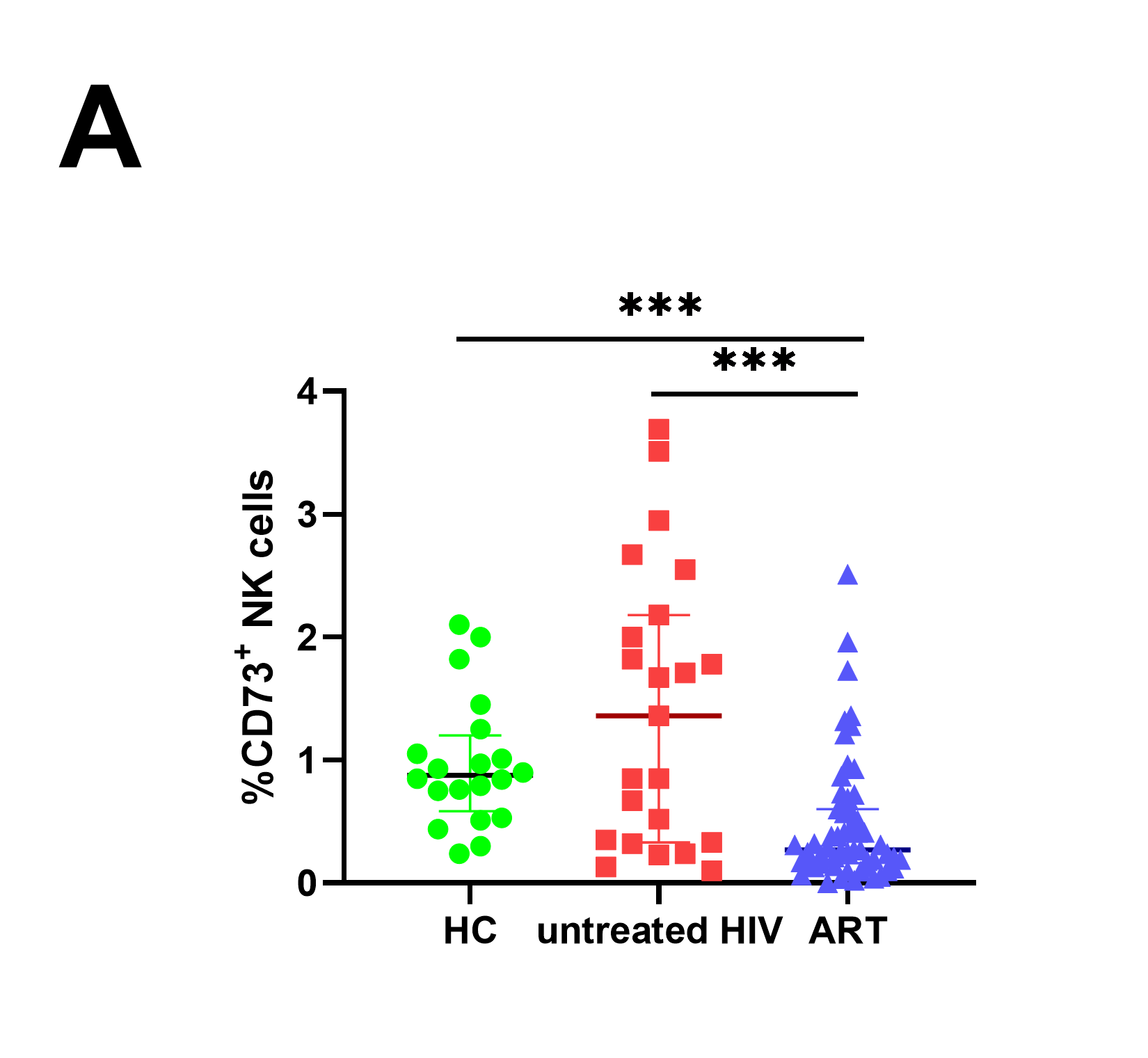

Supplement: Supplementary Figure 1 — The expression of CD73 on NK, CD3+CD4- T and CD3+CD4- T cells in HC, untreated HIV, and ART-treated patients. [file Image_1.tif]
